# Calculated initial parenteral treatment of bacterial infections: Skin and soft tissue infections

**DOI:** 10.3205/id000055

**Published:** 2020-03-26

**Authors:** Cord Sunderkötter, Karsten Becker, Christian Eckmann, Wolfgang Graninger, Peter Kujath, Helmut Schöfer

**Affiliations:** 1 Universitätsklinik und Poliklinik für Dermatologie und Venerologie, Martin-Luther-Universität Halle-Wittenberg, Halle (Saale), Germany; 2 Institut für Med. Mikrobiologie, Universitätsklinikum Münster, Germany; 3 Klinik für Allgemein-, Viszeral- und Thoraxchirurgie, Klinikum Peine, Germany; 4 Vienna, Austria; 5 Chirurgische Klinik, Medizinische Universität Lübeck, Germany; 6 Klinik für Dermatologie, Venerologie und Allergologie, Universitätsklinikum Frankfurt/Main, Germany

## Abstract

This is the ninth chapter of the guideline “Calculated Parenteral Initial Therapy of Adult Bacterial Disorders – Update 2018” in the 2^nd^ updated version. The German guideline by the Paul-Ehrlich-Gesellschaft für Chemotherapie e.V. (PEG) has been translated to address an international audience.

The chapter contains the first German S2k guidelines for bacterial skin and soft tissue infections. They encompass recommendations on diagnosis and treatment of the defined entities erysipelas (caused by beta-hämolytic streptococci), limited superficial cellulitis (*S. aureus*), severe cellulitis, abscess, complicated skin and soft tissue infections, infections of feet in diabetic patients (“diabetic foot”), necrotizing soft tissue infection and bite injuries.

## Zusammenfassung

Dies ist das neunte Kapitel der von der Paul-Ehrlich-Gesellschaft für Chemotherapie e.V. (PEG) herausgegebenen S2k Leitlinie „Kalkulierte parenterale Initialtherapie bakterieller Erkrankungen bei Erwachsenen – Update 2018“ in der 2. aktualisierten Fassung und es enthält die erste deutschsprachige S2k Leitlinie zu bakteriellen Haut- und Weichgewebeinfektionen.

Sie umfasst Empfehlungen zur Diagnostik und Therapie der darin definierten Entitäten klassisches Erysipel (verursacht durch beta-hämolysierende Streptokokken), begrenzte oberflächliche Phlegmone (*S. aureus*), schwere Phlegmone, Abszess, komplizierte Weichgewebeinfektion, Fußinfektion bei Diabetes mellitus (sog. „diabetischer Fuß“), nekrotisierende Weichgewebeinfektion und Bissverletzungen.

## Introduction

Bacterial skin and soft tissue infections (SSTIs) are among the most common infections worldwide. Many derive from chronic wounds (the incidence of which increases with age), others from small superficial lesions (impetigo or foot mycosis), others from acute trauma or surgical wounds.

The clinical features of SSTIs show a broad spectrum from superficial pyoderma to life-threatening myonecrosis with high mortality [1]. In order to classify this multitude of diseases, in 1990 the British microbiologist Kingston proposed a classification into three degrees of severity, the decisive criterion being the necessity or urgency of surgical intervention [2].

Another clinical definition that was relevant for approval until a few years ago was that of “complicated” skin and soft tissue infections (cSSTIs). It was, as it were, an inclusion criterion for clinical antibiotic studies and according to the US Food and Drug Administration (FDA) was present when there was a need for major surgical intervention due to a spread to fascia (severe phlegmon), when more than 3% of the body surface area were affected or when a severe underlying disease or other condition was present which impeded response to treatment (see Table 1 (Tab. 1)) [3].

Since 2013 the following inclusion and progression criteria have been recommended by the FDA, in particular for studies under the heading “acute bacterial skin and skin structure infections” (ABSSSI) [4], [5]. These include “cellulitis” and erysipelas, wound infections and major cutaneous abscesses, which have a minimum spread of erythema, swelling and induration or infiltration of 75 cm^2^. 

Furthermore, in SSTIs it must be taken into account that it may be a local or a diffusely spreading infection with a general reaction [6]. The depth of an infections with spread to the subcutaneous tissue, the fascia or the musculature must also be considered. 

The non-congruent use of entity terms in German-speaking and Anglo-Saxon literature and the lack of generally accepted definitions are problematic for evidence-based recommendations (see below: “cellulitis” term and newly introduced definition of “limited phlegmon”). We therefore discuss the definitions used by us, such as that of an infected wound (without soft tissue infection) versus erysipelas versus limited phlegmon versus severe phlegmon versus necrotizing soft tissue infections [7], [8], [9], [10], [11].

In cases of severe and complicated SSTIs, if initially calculated treatment fails, the next step should be targeted treatment once the pathogens have been identified and tested for sensitivity. The prerequisite for this is the extraction of smears, aspirates or, if possible, correctly taken tissue samples prior to the start of antibiotic treatment [7], [8].

With infections requiring predominantly conservative treatment, i.e. primary cutaneous, superficial bacterial infections, include impetigo contagiosa, erysipelas, limited phlegmon and erysipeloid, sometimes also boils.

Indications for a systemic administration of antibiotics include the diffuse spread of infection in the soft tissue and/or an infection-related general reaction of the body, such as shivering, fever, fatigue, neutrophilic leukocytosis and CRP elevation. In case of fever, three blood culture sets (each both aerobic and anaerobic) should be taken. 

In the 2014 Infectious Diseases Society of America (IDSA) guidelines, the presence of ≥1 or ≥2 signs of the Systemic Inflammatory Response Syndrome (SIRS) are thus interpreted or termed as “moderate” or “severe” SSTIs (“cellulitis”), and as a result the decision about whether to use parenteral treatment and if so, which [12], [13]. The corresponding symptoms, i.e. fever >38°C, hypothermia (<36°C), leukocytosis >12,000/µl or leukopenia <400/µl), tachycardia (>90/min) or tachypnoea (>24 breaths/min) are signs of incipient sepsis. However a SIRS stage is no longer defined, according to the new consensus definition of 2016 [14]. Instead, organ dysfunction is used as the key sign for sepsis, and the so-called “quickSOFA” score determined to allow rapid identification of a possible sepsis or an increased risk of an unfavorable progression in patients suspected of having an infection. It results when ≥2 of the following 3 criteria are applicable (http://www.qsofa.org/) [14] (see chapter 11 [15]):

- low systolic blood pressure (≤100 mmHg),
- increased respiratory rate (≥22 breaths per min) or
- altered mental status (Glasgow Coma Scale <15).

**Indications for parenteral (possibly sequential) antibiotic treatment are generally:**

- severe infection with pronounced systemic signs or signs of incipient sepsis (further criteria for severe infections are explained in detail under respective infections),
- critical localization with risk of serious consequences (for example hands or face),
- presence of corresponding comorbidities (such as circulatory disorders, gastrointestinal absorption disorders),
- relevant immunosuppression.

For many antibiotics, the dosage recommendations must be increased to achieve adequate efficacy, especially in critically ill patients. Critically ill patients are often not included in approval studies. However, in these patients altered volume distribution and clearance lead to systemic reactions with changes in pharmacodynamics and pharmacokinetics. Therefore beta-lactams especially should and can be used in critically ill patients in very high doses (including piperacillin/tazobactam, ceftazidime, cefepime, meropenem), especially initially [16], [17].

Therefore, in Table 2 (Tab. 2) and in some places in the text, in addition to the authorized doses we have also recommended higher doses. The higher doses should be considered if there are signs of sepsis [14] or signs consistent with the former SIRS definition (fever [body temperature >38°C] or hypothermia [body temperature <36°C], leukocytosis [leukocytes >12,000/µl] or leukopenia [leukocytes <400/µl], tachycardia [heart rate >90/min] or tachypnoea [>24 breaths/min]) or in case of insufficient response to otherwise empirically effective antibiotics.

Regarding critically ill patients we also refer to the dosage recommendations in chapter 11 [15].

## Treatment of selected bacterial skin and soft tissue infections

The following are the recommendations for parenteral antibiotic treatment of selected SSTIs. Indications for oral administration (for example erysipelas prophylaxis) are mentioned or reference is made to PEG recommendations for oral therapy [9]. 

Recommendations for the calculated treatment in case of suspected MRSA are summarized in a separate section.

### Impetigo and ecthyma 

Parenteral antibiotics are not required for treatment of impetigo and ecthyma (the deep and ulcerating form of impetigo).

### Boils and carbuncles

The most common pathogen is *Staphylococcus aureus*. In many cases in the USA but also in Germany, these can be caused by PVL-positive MSSA or MRSA (usually called “community-acquired MRSA” [CA-MRSA]). Boils and carbuncles should be lanced if they are adequately abscessed (“matured”) [18]. Maturation can be promoted through ointments containing ichthyol or warm moist compresses (expert opinion). Antibiotics are also usually indicated additionally if the surrounding soft tissue is noticeably reddened and indurated or if systemic symptoms persist (see above) or if there are larger, not yet clearly abscessed (“matured”) boils on the face or other vulnerable regions, (for an overview see [11]).

Localization in the central facial region is an indication for *rapid parenteral* administration of antibiotics, to reduce the risk of orbital cellulitis, cavernous sinus thrombosis or meningitis. 

*Drugs of first choice:* Cefazolin 3x 1 g/d i.v. or flucloxacillin 3x 1 g/d i.v. (higher plasma protein binding) (expert opinion).

*Drugs of second choice:* Cefuroxime i.v. (3x 1.5 g) or clindamycin i.v. (in the case of a pure abscess 3x 0.6 g [according to package insert], with phlegmonous spreading 4x 0.6 g or 3x 0.9 g [according to the package insert] up to 3x 1.2 g [expert opinion]) [18], [19].

*In case of penicillin allergy:* Clindamycin (dose see above).

The duration of treatment should ideally be 5–7 days [18], [20].

If boils develop extremely rapidly in otherwise healthy patients, expand rapidly or if necroses rapidly form or recur, PVL-producing *Staphylococcus aureus* should be considered as a possible cause (further steps below in the section on MRSA).

### Furunculosis

The treatment is similar to that of boils, supplemented by restoration of pathogen reservoirs (for example PVL-positive *Staphylococcus aureus*) and treatment of underlying diseases (diabetes control) [19].

### Abscesses

Cutaneous abscesses are encapsulated pus-filled cavities in the dermis and subcutis resulting from tissue destruction by granulocytes and bacterial enzymes, which present clinically bulging, fluctuating, dark-red, painful, overheated swellings under mostly intact epidermis. Depending on their origin they are caused by one or more bacterial species. The pathogens (mainly *Staphylococcus aureus* but also hemolysing streptococci, and especially Enterobacteriaceae) usually originate from the resident or transient skin flora (microbiota) and, with penetrating lesions, also from the invading object. Abscesses can constitute a high risk of complications and belong to the type of compartments which are generally difficult to reach with antibiotics.

*Treatment of first choice:* Incision and drainage, surgical care.

*Comment on the evidence:* According to studies and systematic reviews, incision and drainage is the most important and primarily effective treatment of abscesses, also to prevent spreading. Depending on the size of the abscess, incision and drainage may be sufficient even if there is an infection with CA-MRSA [18], [21], [22]. Note: Shortly before going to print, a study from the US found a significantly better cure rate for *Staphylococcus aureus* abscesses ≤5 cm^2^ (MSSA and MRSA) when, after drainage for 7–10 days, clindamycin or trimethoprim/sulfamethoxazole was given (83.1% and 81.7% compared to 68.9% for placebo). After administration of clindamycin, fewer relapses occurred within one month (6.8%) than after trimethoprim/sulfamethoxazole (13.5%) or placebo (12.4%) but there were also more adverse events (21.9% versus 11.1% or 12.5%). However, the study did not indicate how high the proportion of PVL-formers was, although PVL-positive MRSA (USA300) is endemic in the US. In addition, the duration of treatment was unusually long [23]. The results are not directly transferable to the situation in Central Europe and thus did not alter our recommendations.

After incision, depending on the size of the abscess, consideration may be given to packing the abscess cavity with hydrofiber silver dressing, which has been shown to be superior to iodophorm gauze in terms of healing time and pain [24]. No general rule can be inferred from this, since there is no controlled study that demonstrates the benefit of packing at all and because other packing materials were more painful than a simple sterile cover [25]. The most important aspect is to ensure continuous outflow. 

Indications for the additional administration of antibiotics are found in cases of:

- localization on the face, hands or genito-anal area,
- chambered abscesses or other factors complicating adequate drainage (sufficient drainage should, whenever possible, take precedence and not be replaced by administration of antibiotics),
- recurrent abscesses [19],
- diffuse spreading into the soft tissues (phlegmon), particularly if a) the surrounding soft tissue is clearly reddened and indurated or systemic symptoms persist (see above); and b) the process is not already clearly abscessed (“matured”);
- immunosuppression,
- risk factors in the sense of a complicated SSTI,
- systemic symptoms (see above).

Since abscesses are difficult to target for antibiotics, especially antibiotics with good cell membrane penetration should be used (clindamycin and also some fluoroquinolones [especially moxifloxacin]). Treatment can be optimized after pathogen detection and sensitivity testing. Following normalization of temperature, general symptoms, CRP, and leukocytosis <8,000/µl antibiotic treatment may be terminated. Five days of treatment may suffice [20]. If early-stage immature abscesses cannot be distinguished from a limited phlegmon, initial treatment should match that of limited phlegmons.

In case of indications for antibiotic treatment but without the need for parenteral administration, after successful drainage oral therapy for 5–7 days may be sufficient: Cefadroxil (2x 1 g p.o., maximum 4 g/d) or cefalexin (3x 1 g p.o.) (Study in children: [18]), in deep abscesses clindamycin (3x 0.6 g/d) (good tissue penetration and activity against anaerobes) [18], [19].

- *In case of indication for parenteral administration and suspected exclusively Gram-positive aerobic pathogens:* Cefazolin 4x 0.5 g [26] or 2x 1 g (in Gram-negative pathogens up to 2x 2 g [package insert]) or flucloxacillin (3x 1 g or 4x 1 g [package insert]). For the treatment of severe, life-threatening infections (based on measured tissue levels [26]), cefazolin is given higher doses of 3x 1–2 g/d (package insert indicates a maximum daily dose of 6 g, for life-threatening infections a maximum of 12 g) and for flucloxacillin 12 g per day is recommended (expert opinion) (maximum dose according to package insert).
- *In case of deep abscesses:* Clindamycin (3x 0.6 g/d (package insert) or 4x 0.3 g/d for at least 5 days (clinical study on oral administration in infected gingival and odontogenic, drained abscesses [20]). For severe skin infections higher doses are recommended (for example clindamycin 3x 0.9 g/d, 4x 0.6 g/d or 3x 1.2 g/d (expert opinion), the maximum dose according to the package insert is 4.8 g/d.
  Although moxifloxacin was slightly superior to clindamycin in this study, it is not recommended to the same extent because of its other properties.
- *For superficial abscesses possibly caused by Gram-negative bacteria:* due to correspondingly high bacterial contamination at the entry portal: Cefuroxime (3x 750–1,500 mg/d), second choice cefazolin at a dose of at least 2x 2 g/d).
- *If the abscess is pronounced or phlegmonous and infection with anaerobes or Gram-negative pathogens is likely*, i.e. in penetrating traumas with contaminated objects, bacteria-rich areas, wound infections after operations in the area of the axilla, the intestine, the perineal area or the female genital tract:
  - First choice: Ampicillin/sulbactam [27] or amoxicillin/clavulanic acid (3–4x 1.2 g) (study on complicated SSTIs for example [28]).
  - Second choice: Piperacillin/tazobactam (due to the very broad spectrum), followed by oral administration of amoxicillin/clavulanic acid [29].
  - In case of penicillin allergy: Ciprofloxacin plus metronidazole (no study, expert opinion); with a lower Recommendation Grade: moxifloxacin.
- *In case of recurrent abscesses additionally:* a) diagnostics to exclude pilonidal sinus, acne inversa, foreign bodies and infection with PVL-positive *Staphylococcus aureus* (CA-MRSA or MSSA); exclusion of neutrophile disorders (only if relapses began in childhood); b) if necessary (in the case of *Staphylococcus aureus*) a 5-day local decolonization with nasal mupirocin and daily whole-body washing (for instance with chlorhexidine, octenisan) as well as daily change of personally used items (towels and similar); however, the evidence for this is weak [12].
- *In case of suspected* CA-MRSA (see also separate section on MRSA):
  - *First choice:* (based on existing studies in the USA with mainly CA-MRSA): oral administration of trimethoprim/sulfamethoxazole (2x 160/800 mg/day, effective against CA-MRSA, but also against hospital-associated MRSA [HA-MRSA] and streptococci) [30], [31], [32], [33] or clindamycin (also parenteral [34], also effective against streptococci).
  - *Second choice:* oral doxycycline [12], [35]. Further procedure according to antibiogram as well as recommendations for MRSA decolonization [36].

*Comment on the evidence:* There are only a few studies for the indications mentioned here and for the selection of a systemic antibiotic treatment and these usually include phlegmons [30], [31], [34].

In the PEG resistance study in 2013, all HA-MRSA (n=75) and *Streptococcus pyogenes* isolates (n=246) were cotrimoxazole-sensitive [32], [33].

Further controlled randomized studies on uncomplicated purulent skin infections are available for oral antibiotics:

- Clindamycin and cephalexin for 7 days for the treatment of uncomplicated skin and soft tissue infections (SSTIs) in children in the US, mostly caused by CA-MRSA: 97% cure with cephalexin and 94% with clindamycin after 7 days (difference not significant) [18].
- Placebo-controlled or direct comparative studies on uncomplicated SSTIs (“abscesses >5 cm” or so-called “cellulitis” in the sense of a non-festering infection) were last done on oral antibiotics in the USA. They therefore related to a population with a high prevalence of PVL-positive MRSA (ST-8 or USA300). One study showed that oral trimethoprim/sulfamethoxazole (2x 320/1,600 mg per day for 7 days) in drained abscesses increased the healing rate versus placebos (92.9% vs. 85.7%) and the number of repeated drainages or further abscesses decreased [31]; the latter was also confirmed by an older study which, however, did not show any better healing of the drained abscess [30]. A further comparative study on uncomplicated skin infections (in the sense of abscesses >5 cm or so-called “cellulitis”) showed no significant difference between trimethoprim/sulfamethoxazole (2x 160/800 mg per day p.o.) and clindamycin (3x 300 mg per day p.o.) [34]. In a US study, no significant difference between the two o.a. daily doses of trimethoprim/sulfamethoxazole [35].

The IDSA guidelines recommend antibiotic treatment, which is effective against both PVL-positive MRSA and streptococci, for the calculated treatment of abscesses: clindamycin or trimethoprim/sulfamethoxazole plus a cephalosporin [12]. In our experience in Germany, Austria and Switzerland, streptococci are rarely the main cause of abscesses. In German-speaking countries, where there is no high prevalence of CA-MRSA but at best PVL-positive MSSA [37], in cases of uncomplicated limited infections isoxazolylpenicillins (flucloxacillin in Germany) are thus very effective against *Staphylococcus aureus* and exercise a relatively low selection pressure (expert opinion). Flucloxacillin has a higher plasma protein binding than cephalosporins, a genetically determined risk of hepatopathy (and an adverse interaction, for example with methotrexate). They should therefore not be given for more than 14 days. 

Further comments on the evidence can be found in the section “Limited Phlegmons”.

### Erysipeloid (“swine erysipelas”)

The pathogen is *Erysipelothrix rhusiopathiae*. The treatment of choice for local infections and the rare systemic spread (with fever, endocarditis or arthritis) is the oral administration of penicillin. For patients with a penicillin allergy, clindamycin or fluoroquinolones are possible candidates. Note the resistance of erysipelothrix to glycopeptides and sulfonamides.

### Erysipelas (“St. Anthony’s fire”)

Classic erysipelas is an acutely bacterial, non-purulent infection of the dermis, including the lymphatic spaces and vessels, involving significant inflammation and usually derives from small entry sites; beta-hemolytic streptococci, mostly group A (*Streptococcus pyogenes*) and more rarely groups B, C and G [38], [39] are usually seen as the underlying pathogens. Detecting these in the tissue or at the entry sites in culture often fails [summarized in [39]], [Sunderkötter, Becker, et al., manuscript in preparation]. This definition is clinically relevant because beta-hemolytic streptococci are penicillin-sensitive, making penicillin the treatment of choice.

Entry sites are usually small lesions (for example mycosis between the toes, impetigo contagiosa, wounds). The characteristic symptoms and parameters which, amongst others, distinguish it from limited phlegmons are:

1. an acute, overheated, slightly painful, bright red erythema with a shiny surface, sharply defined margins and tongue-shaped processes, usually starting a few centimeters away from the entry site,
2. systemic inflammatory reaction right from the start, consisting of fever or at least shivering, rarely chills, as well as
3. increased ESR, increased CRP and/or neutrophilia.

Depending on the toxin production of the bacteria and the patient’s inflammatory reaction, blisters and bleeding may occur. Transition to bacteremia and sepsis is possible. Most common sites are the lower legs or the face. In principle, however, any skin area can be affected. There are swellings of draining lymph nodes and occasionally visible lymphangitis. 

The diagnosis becomes more difficult only if the skin is significantly altered, for example by chronic stasis or other dermatoses or in facial infections, as the therapeutically relevant distinction from limited phlegmons (see below) is not always possible.

- In uncomplicated erysipelas of otherwise healthy adults, oral treatment with phenoxymethylpenicillin (penicillin V), 3x 1.2–1.5 million IU/d for 7–14 days, is sufficient.
- Parenteral antibiotic treatment of erysipelas is indicated *in complicated erysipelas* in the form of hemorrhagic, necrotizing or blistering erysipelas and/or localization of the face and other *indications for systemic antibiotics* (such as venous or arterial circulatory disorders, gastrointestinal absorption disorders). Relative indications for parenteral therapy are clear systemic signs such as fever, leukocytosis or neutrophilia and a CRP increase. In patients with immunocompetence, oral treatment may be sufficient or a quick switch to oral treatment (sequential therapy) may be possible.
  *Drugs of choice:* Penicillin G i.v. 3x 10 million IU/day for 7–10 days or for about 5–7 days with subsequent oral administration of penicillin V (3x 1.2–1.5 million IU/day). (Additional comments on the doses of penicillin G and penicillin V in the treatment of the complicated erysipelas – EUCAST has recently used the following doses as the basis for “penicillin-sensitive” evaluation: Phenoxymethylpenicillin: 3–4x 0.5–2 g orally and benzylpenicillin: as high dose 4–6x, 1.2 g (corresponding to 4–6x 2 Mio IU). In the authors’ experience, the dosage 3x 10 million IU/day in erysipelas has proven effective for decades and in daily hospital routines three administrations of a high dose have proven more practical and reliable compared to four administrations (thus including one nightly administration). Those studies included in a Cochrane Review [40] give doses of 3x 3 g (3x 5 million IU), 8x 2.5 million IU until free from fever, 6x 18 [sic] million IU until free from fever and in the IDSA guideline 4–6x 2–4 million IU [12]. The duration of treatment is at least 7–10 days, depending on the severity; included are 1–2 days of treatment beyond the reduction of clinical symptoms to minimize the risk of streptococcus persistence in tissue [9], [38], [40].
  Treatment should always include any possible entry sites (for example interdigital mycosis).
- If a limited phlegmon (see below) cannot be excluded with sufficient certainty, for example on pre-damaged skin, we initially recommend a primary application of penicillin G i.v. (3x 10 million IU/day) but in the absence of response within 1–3 days, a switch to flucloxacillin or cefuroxime i.v. (3x 0.75 g to 3x 1.5 g/d) [26], [40], [41], [42].
  In erysipelas-like infections of the face, even on previously unaltered skin, infections caused by *Staphylococcus aureus* or *Haemophilus influenzae* may be clinically very similar to erysipelas. Therefore, for practical reasons, we recommend either the administration of cefuroxime or if observation is possible (for example under in-patient treatment) only initial administration of penicillin, then switching to cefuroxime after 1–3 days if the response is inadequate (cefazolin has no effect on *Haemophilus*) (adjusted based on recommendations and meta-analysis [12], [43]). *Reason:* Patients with soft facial tissue infections or suspected phlegmons should be hospitalized and seen on a daily basis so that penicillin, the most effective antibiotic with the least side-effects, can be used as the primary antibiotic in cases of streptococcal infection but if necessary changed to cefuroxime in time.

In case of penicillin allergy:

- *First choice:* Clindamycin (3x 0.3 g/d to 3x 0.6 g/d for 7–10 days)
- *Second choice:* Clarithromycin (2x 0.5 g/d i.v.) or roxithromycin 1x 0.3 g/d p.o. (a form for intravenous administration has not been licensed in Germany).
- *Third choice:* Moxifloxacin (1x 400 mg)

*Comment on the evidence:* Penicillin is very well suited for erysipelas treatment and its oral bioavailability comparable to its parenteral administration (small controlled comparative study) [38]. Aminopenicillins are less active against streptococci than penicillin and cephalosporins of groups 1 and 2 and thus, considering their adverse effects, less well suited for targeted treatment.

In case of a penicillin allergy, clindamycin or a macrolide is recommended, which has been shown to be effective according to studies and meta-analyzes that did not strictly distinguish between erysipelas and limited phlegmons [34], [43].

The newer macrolides have a great potential for interaction, for example with antiarrhythmic drugs. Moxifloxacin shows an unfavorable risk-benefit ratio (see the section on limited phlegmons), especially in elderly patients with classic erysipelas. The approved dose of moxifloxacin (400 mg/d) may not always be sufficient in overweight or large patients. In this situation, a dose of 2x 400 mg/d can be considered as an off-label treatment for the first two days. There are no appropriate studies on parenteral administration of clarithromycin and regarding oral administration, only studies on children with skin and soft tissue infections [44], [45]; the data is better regarding the efficacy and safety of clarithromycin in the treatment of skin infections with (non-tuberculous) mycobacteria [46]. 

Flucloxacillin has low MIC_90_ values for *Streptococcus pyogenes* (0.064 mg/l) and group C and G streptococci (0.25 mg/l) (EUCAST Antimicrobial wild type distributions of microorganisms; https://mic.eucast.org/Eucast2/) and is effective in cases of “cellulite” sensitivity [47] but showed a higher rate of adverse side effects compared to penicillin G. The use of flucloxacillin should be limited to the treatment of infections by penicillinase-producing, methicillin-sensitive staphylococci. Thus, we do not recommend flucloxacillin as the first-choice treatment for erysipelas. 

With regard to the duration of treatment, different treatment regimens were used in the respective studies. Often it was either 7–10 or 10–14 days. In a study on the treatment of “cellulitis” (uncomplicated SSTI without separation between limited phlegmons and classic erysipelas) with levofloxacin, there was no difference between the success rates of 5– and 10–day treatments [48] but due to the potential risk of recurrence, we recommend treatment duration of at least 7 days (in the case of edema or PAOD at least 10 days). For the same reason, treatment with clindamycin should also be limited to 10 days to minimize the risk of *Clostridium difficile* colitis. 

### Chronic recurrent erysipelas

Inadequate treatment of erysipelas or lack of treatment of the entry site leads to recurrences. Repeated episodes of erysipelas (recurrent erysipelas) in turn cause increasing irreversible damage to the lymphatic vessels and result in serum-rich edema, which creates the conditions for renewed recurrences. Traditionally, recurrence prophylaxis is recommended after 3–4 recurrences per year [12] but according to recent studies, it has proven useful even after the first recurrence [49]. A common cause of recurrent erysipelas-like erythema in (lymph) edema is recurrent acute stasis dermatitis (hypodermatitis) or neutrophilic dermatitis on lymphedema, which may also be associated with mildly elevated CRP and should be excluded to avoid unnecessary administration of antibiotics.

*Treatment of choice:* Initially parenteral treatment of the acute erysipelas recurrence (see above), i.e. penicillin G. 3x 10 million IU/day i.v. for 7–14 days; thereafter prophylactic long-term treatment with phenoxymethylpenicillin (penicillin V) 2x 250 mg/d or 2x 0.425 million IU/d for 12 months [49], [50]. This dosage is difficult to achieve in Germany because most available tablets contain 1.2 or 1.5 million IU, corresponding to 708.0 or 885.0 mg phenoxymethylpenicillin. While a syrup is available, this is not as practical. If the tablet has a score line (division aid) and after consultation with the pharmacist is deemed divisible, 2 half tablets of 1.2 million IU can be taken, however, there would be no evidence for the effect and tolerability of this slightly higher dose.

Indication for parenteral prophylactic treatment is a lack of compliance. It consists of prophylaxis with depot penicillin (benzathine benzylpenicillin 2.4 million U i.m. every 2–3 weeks [51] (meta-analysis [50]). If there is no relapse after about 6 months, the interval can be extended.

*Recommendations for cases of penicillin allergy:* clarithromycin 250 mg/d p.o. for 12 months. There is no evidence for the recommended dose; it is derived from a case series with erythromycin and corresponds to the low prophylactic erythromycin dose used there; erythromycin itself is no longer recommended as an antibiotic because of its unfavorable absorption rate and adverse effects.

*Comment on the evidence:* The corresponding studies [49], [50], [51] have already been mentioned above.

### Limited phlegmons (limited soft tissue infection, for example in chronic wounds)

A limited phlegmon is a partially purulent infection of the dermis and subcutis, which is neither a (streptococcus-related) erysipelas nor a purulent-necrotic infection reaching the fascia (severe phlegmon). It usually requires no surgical intervention but antimicrobial treatment instead.

A limited phlegmon usually occurs around a larger wound (but can be distinguished from wound infection or wound colonization) and is often caused by *Staphylococcus aureus* in immunocompetent patients, even if the entry site is multibacterially populated or infected with other pathogens. Despite infection of the soft tissue, it does not necessarily meet the minimum size requirement of 75 cm^2^ for acute bacterial skin and skin structure infections (ABSSSI). The term “limited phlegmon” was first used in the 6a/6b Microbiological-Infectious Quality Standards of the German Society for Hygiene and Microbiology and the recommendations of the Paul-Ehrlich-Gesellschaft for the rational use of oral antibiotics in skin and soft tissue infections [7], [8], [9], [10], [11]. In English, the term cellulitis is sometimes used in this sense but similarly often as a generic term for erysipelas and the limited soft tissue infection referred to here [13].

The clinical criteria include overheated, edematous, painful, dark or livid redness or doughy swelling around an entry site (ulcer, wound). The lesion is usually of a darker or even livid red shade as well as duller and less defined than in classic erysipelas. Initially, systemic signs of infection such as leukocytosis with neutrophilia, fever, increase in ESR or CRP are absent. If *Staphylococcus aureus* is isolated from the entry site, it is usually also the causative agent in the infected dermis tissue, regardless of whether other colonizing or contaminating microorganisms, such as representatives of skin microbiota or Gram-negative bacteria from the intestinal microbiota are isolated in the wound swab (Sunderkötter, Becker, et al. unpublished results). 

In cases of more severe diseases or other impairments of the immune system (peripheral arterial occlusive disease, poorly controlled diabetes mellitus, immunosuppression, including neutropenia), other relevant bacteria can also be isolated from the soft tissue. But in this case, it is usually no longer a limited but rather a severe phlegmon or complicated SSTI. 

*Indications for the parenteral (instead of oral), if necessary sequential, antibiotics *(see above)* here would be*

- the above-mentioned criteria, such as systemic signs of infection; or
- limited blood flow or absorption,
- superficially extensive infections,
- a transition to deeper, severe phlegmons,
- localization at the flexor tendons or in the face.

The higher dose options referred to in the following paragraphs should be considered when signs of sepsis or the former SIRS definition are present (i.e. fever [body temperature >38°C] or hypothermia [body temperature <36°C], leukocytosis [ leucocytes >12,000/µl] or leukopenia [leukocytes <400/µl], tachycardia [heart rate >90/min] or tachypnoea [>24 breaths/min]) or in case of insufficient response after empirical administration of otherwise effective antibiotics.

- *First choice drugs for uncomplicated infections:* Cefazolin 4x 0.5 g [27] or 2x 1 g (in Gram-negative pathogens up to 2x 2 g [package insert]) or flucloxacillin (3x 1 g or 4x 1 g according to package insert). For Cefazolin, following measurement of tissue levels [26], higher doses of 3x 1–2 g/d (the package insert indicates a maximum daily dose of 6 g, for life-threatening infections no greater than 12 g) and for flucloxacillin 12 g per day (maximum dose according to package insert) are recommended for treatment of severe, life-threatening infections (expert opinion).
- *Second choice drugs for uncomplicated infections:* Clindamycin if the area around the entry site is heavily contaminated or colonized with Gram-negative pathogens: Cefuroxime (3x 1.5 g/day i.v.) [18], [19].

In case of penicillin allergy:

- 1. choice: Clindamycin (3x 0.9 g/d)
- 2. choice: oral clarithromycin 2x 0.5 g/d for 7–10 days [18], [19] (roxithromycin 1x 0.3 g/d only approved for oral administration in Germany)

It can be deduced from studies on infections with abscess formation that moxifloxacin is similarly effective as clindamycin but it is not recommended by us to the same extent because of its other properties.

*Comment on the evidence:* Since there are no recognized criteria for this group of limited SSTIs (“limited phlegmons”), there are hardly any studies available with corresponding inclusion criteria, in contrast to the situation with the so-called complicated SSTIs according to the FDA definition but which usually do not apply to limited phlegmons.

Studies from recent years, which partly based their criteria on the new definitions for acute SSTIs, also often involve SSTIs which are more complicated or severe phlegmons; or these studies do not differentiate clearly between abscesses (> 5 cm^2^ area) and “cellulitis” in the sense of a non-festering infection or involve US populations with their high CA-MRSA prevalence. 

In a Cochrane analysis on “cellulitis and erysipelas” of 2010 [40], no conclusive recommendations could be made either for erysipelas or for limited SSTIs grouped under “cellulitis”. Another meta-analysis mentions erysipelas and “cellulitis” in the title but does not further distinguish between them in the text and describes them as infections which are clinically indistinguishable and mostly caused by *Streptococcus pyogenes* or *Staphylococcus aureus* (or by MRSA in corresponding endemic areas). Fifteen studies were compared in which various beta-lactam antibiotics (penicillin, cloxacillin, flucloxacillin, dicloxacillin, cephalexin, cefprozil, cefaclor) as well as macrolides and lincosamides (clindamycin) were used. The authors concluded that the efficacy and tolerability of the substances are comparable and sufficient with respect to the common spectrum of pathogens but that in regions with high CA-MRSA prevalence, macrolides or lincosamides should be preferred to the beta-lactam antibiotics mentioned [43]. According to a 2006 meta-analysis, beta-lactam antibiotics (without group 3 cephalosporins) were as effective in treating mild to moderate infections as fluoroquinolones but should be preferred for their lower rate of adverse events [52].

The IDSA guidelines recommend antibiotics which target methicillin-sensitive *Staphylococcus aureus* (MSSA) for the calculated treatment of non-festering “cellulitis” with systemic signs (in our definition, erysipelas and to a certain extent limited phlegmons): Flucloxacillin 1–2 g every 4 hrs i.v., cefazolin 1–2 g every 8 hrs i.v., clindamycin 600 mg every 8 hrs i.v., dicloxacillin 500 mg every 6 hrs p.o., cephalexin 500 mg every 4 hrs p.o., doxycycline or minocycline 100 mg all 12 hrs p.o., trimethoprim/sulfamethoxazole 160 mg/800 mg every 12 hrs p.o.

In the case of “cellulitis” associated with penetrating trauma, drug abuse and detection of MRSA in another infection or in the nose, vancomycin and antibiotics which are effective against MRSA and Streptococci, are recommended as in cases of severe SSTIs (severe phlegmons): vancomycin 30 mg/kg/d i.v. in 2 doses (also the drug of choice in cases of penicillin allergy), linezolid 600 mg every 12 hrs i.v. (or 600 mg every 12 hrs p.o.), clindamycin 600 mg every 8 hrs i.v. (or 300–450 mg every 6 hrs p.o.), daptomycin 4 mg/kg every 24 hrs i.v., ceftaroline 600 mg every 12 hrs i.v. but also doxycycline, minocycline and trimethoprim/sulfamethoxazole [12], [13].

Our recommendations differ in that we take a more differentiated approach to the indications. 

5 days are generally recommended as duration of treatment, since a duration of 10 days showed no advantage [50], duration should only be extended in the absence of improvement [12].

Placebo-controlled or direct comparative studies on uncomplicated SSTIs (“abscesses >5 cm” or so-called “cellulitis” in the sense of a non-festering infection) were last done on oral antibiotics in the USA. They therefore relate to a population with a high prevalence of PVL-positive MRSA. They are described in the section “Abscesses”.

In a comparative study on uncomplicated skin infections (in the sense of abscesses >5 cm or so-called “cellulitis”) there was no significant difference between trimethoprim/sulfamethoxazole (2x 160/800 mg per day p.o.) and clindamycin (3x 300 mg per day p.o.) [34]. As previously mentioned, no significant difference was found in a US study [35] between a low daily dose (2x 160/800 mg per day p.o.) of trimethoprim/sulfamethoxazole and a high daily dose (2x 320/1,600 mg per day p.o.).

A randomized double-blind study with a relatively small sample size of 58 in-patients, compared parenteral administration of ampicillin (4x 1 g) plus sulbactam (4x 0.5 g) with that of cefoxitin (4x 1 g) for treatment of skin and skin appendage infections, and cefazolin (4x 0.5g) in cases of “cellulitis” (which here most likely refers to “limited phlegmons”). In “cellulitis” treatment success (cure or significant improvement) was achieved with ampicillin/sulbactam and cefazolin in 100% and 91.7% of cases within 7.7 and 7.2 days and in the other SSTIs, treatment success with ampicillin/sulbactam and cefoxitin was achieved in 80% and 64.7% of in-patient cases within 7.7 and 9.4 days respectively. Overall, there were no significant differences in efficacy and adverse effects between the treatment groups [27].

A comparison between oral cefalexin and clindamycin in children with uncomplicated, to some extent purulent SSTIs and high prevalence of CA-MRSA also showed no significant difference [18].

As there is no high prevalence of CA-MRSA in the German-speaking countries, isoxazolylpenicillins are also recommended for the above-mentioned reasons (see section on “Abscesses”). 

Flucloxacillin also has low MIC_90_ levels for *Streptococcus pyogenes* (see section on “Erysipelas”). In a randomized study, intravenous monotherapy with flucloxacillin (4x 1 g i.v.) in patients with “cellulitis” on the lower legs was as effective as the combination therapy of flucloxacillin with benzylpenicillin (1x 1.2 g i.v.) [47]. In two retrospective studies cefazolin was compared to oxacillin (not flucloxacillin) in cases of complicated bacteremia caused by MSSA [53], [54]. The efficacy of both antibiotics was considered to be comparable but the rate of adverse events in the oxacillin group was higher in one [54] and comparable to cefazolin in the study of higher levels of soft tissue infections [53]. It is conceivable that the efficacy and safety profile of flucloxacillin is slightly better than that of oxacillin.

Compared to cefazolin, cefuroxime has a broader – albeit limited – action spectrum in the Gram-negative range (including *Haemophilus influenzae*) and also shows activity against *Staphylococcus aureus*, which is lower than that of cefazolin and isoxazolylpenicillins. *Enterobacter* spp., *Citrobacter* spp., *Morganella morganii* and *Proteus vulgaris* are often resistant but these usually are not relevant in limited, non-complicated soft tissue infections. Cefuroxime should not be used orally because of its comparatively low bioavailability. Although frequently administered parenterally in SSTIs, there is no good study in adults. A randomized, prospective, comparative study in children with SSTIs between cefuroxime (50–100 mg/kg/d divided into 3 or 4 daily doses) and ampicillin/sulbactam (150–300 mg/kg/d divided into 4 daily doses) for a maximum of 14 days showed no significant difference in efficacy [55]. As regards the dosage of cefazolin, according to the results of a study which determined tissue levels of cefazolin, a dosage of 3x 1 g/d i.v. in *Staphylococcus aureus* infections appears to be sufficient in most cases, while infections of Enterobacteriaceae probably require at least 3x 2 g [26]. For other antibiotics, by analogy to infections with abscess formation in other tissues, we have additionally indicated higher doses than the standard dosages referred to in publications or package inserts in Table 1 (Tab. 1), for example for clindamycin from 4x 0.3 g [21], [36] or 3x 0.9 g (package insert) to 3x 1.2 g or 3x 1.8 g (expert opinion).

Currently, studies are being carried out with cephalexin plus trimethoprim/sulfamethoxazole versus placebo (NCT00676130) and with oral flucloxacillin plus phenoxymethylpenicillin versus flucloxacillin alone in “cellulitis” (most likely referring to erysipelas and limited phlegmons and possibly infections with abscess formation) (“Oral flucloxacillin and phenoxymethylpenicillin versus flucloxacillin alone for the emergency department out-patient treatment of cellulitis”, EudraCT Number 2008-006151-42) [56], [57].

In cases of penicillin allergy, clindamycin is the drug of choice. If there is no clinical improvement within 2–3 days and an antibiogram identifying the pathogen is not yet available, a fluoroquinolone (moxifloxacin) may be given, especially if Gram-negative bacteria are a consideration. For mild infections, initial treatment with clarithromycin would also be a possibility (for the limited data situation, see above [44], [45]).

Resistance to non-beta-lactam antibiotics also occurs in *Staphylococcus aureus* isolates (especially MRSA) of out-patients. However, in Germany, unlike in the US, HA-MRSA (also known as livestock-associated MRSA [LA-MRSA]) rather than CA-MRSA is prevalent in areas with intensive animal husbandry. In the 2013 PEG resistance study, the following resistance rates were determined for out-patient isolates: Inducible and constitutive resistance to clindamycin, 7.3% (MSSA, n=343) and 43.3% (MRSA, n=30), respectively; clarithromycin, 13.1% (MSSA) and 56.7% (MRSA) and moxifloxacin, 7.6% (MSSA) and 76.7% (MRSA), respectively [33].

### Severe phlegmons (invasive, cross-tissue, usually purulent infection requiring urgent surgical care and/or marked signs of systemic reaction)

Clinically, a “severe phlegmon” manifests – like a “limited phlegmon” – as an overheated, edematous, painful, dark redness or doughy swelling but in additional there is a significant accumulation of pus and possibly even necrosis, crossing to lower levels of soft tissue such as fascia, possibly also muscle layers. There is usually regional lymphadenitis, severe pain and fever and occasionally criteria of the former SIRS definition may occur (see above), especially in the case of inadequate treatment.

*Specimen collection:* Except in instances of infections with abscess formation, IDSA guidelines recommend samples of tissue for cultural detection only for severe infections in antineoplastic chemotherapy, neutropenia, deficiency of cell-mediated immunity, and immersion or bite injuries; in such cases always in combination with blood cultures [12], [13]. The reason for this limitation is the low yield or specificity [13]. In our experience, if sampling is carried out correctly, especially in antibiotic-naive patients, the yield or specificity is sufficient to allow broader recommendations. If an SSTI develops in the direction of a severe phlegmon or complicated SSTI, adequate sample collection is advisable [7], [8], [58]. Other urgent indications for pathogen identification in culture are those limited phlegmons that do not respond to an antibiotic effective against *Staphylococcus aureus* within 2–3 days. These include abscesses, skin infections after surgical interventions or other iatrogenic procedures, infections under immunosuppression with the possibility of more rare pathogens (such as *Cryptococcus* sp. and other fungi) as well as bites or skin rash associated with systemic infections (for example endocarditis, sepsis, rickettsiosis, rat bite fever, and systemic mycosis). 

In general, tissue specimens in their native state are better for pathogen diagnostics than smears because they allow the use of both cultural and nucleic acid detection techniques (for example PCR techniques) and histological examination. This increases sensitivity and specificity. Carefully extracting these is complicated but justified. For this purpose, a tissue spindle about 1 cm in length should be taken from the infected tissue 1–2 cm away from the edge of the wound, down to the subcutis. Prior to this, a reduction of the skin microbiota must be ensured by disinfecting the skin at the sample site. After sterile removal of the tissue sample, it is additionally recommended to separate the upper, epidermal part from the lower dermis and subcutis with another, sterile scalpel so that only the pathogens in the soft tissue are identified. For separation, it is advisable to place the biopsy on a sterile, firm base (not on fleece). Afterwards the tissue sample to be examined is transferred into a sterile transport medium or into a sterile liquid enrichment medium (in particular if small samples are in danger of drying out and/or the possibility of sensitive pathogens) [7], [8]. Their benefits over smears are currently under further investigation in comparative studies. 

For a suitable smear from the entry site, swabs with Dacron tissue should be used for transport media, superficial secretions removed with sterile swabs and fibrinous or necrotic pads removed. Sampling takes place from the wound bed and under the wound margins, if possible from different locations [7], [8]. Flat or spiral-shaped smears appear less suitable because they detect many clinically irrelevant microorganisms contaminating or colonizing the surface.

While procalcitonin is a helpful decision-making aid for determining when to initiate and stop antibiotic treatment for pneumonia or sepsis, there is insufficient data regarding SSTIs at the moment for making clear recommendations [13].

Treatment: Additional measures – treatment of the entry site, the predisposing factors (edema) and comorbidities, propping up the affected area, [if appropriate prophylaxis against thrombosis].

- *First choice drug apart from surgical restoration* of severe phlegmons without treatment to date (for example late treatment of an initially limited phlegmon) and without serious comorbidities
  - cefazolin 4x 0.5 g [27] or 2x 1 g, in case of suspected Gram-negative pathogens up to 2x 2 g; for serious, life-threatening infections) higher doses (3x 2 g/d [26] (the pathogen indicates a maximum of 6 g, for life-threatening infections up to a maximum of 12 g) OR
  - flucloxacillin 3x 1 g or 4x 1 g according to the package insert, in life-threatening infections according to expert opinion up to 12 g daily dose (maximum dose according to the package insert) [10], [27], [53], [59]) OR
  - cefuroxime 3x 1.5 g/day i.v. (package insert) up to 3x 3 g if progression is severe (expert opinion)

In the absence of a response, penicillin allergy or untreated but deeper phlegmons:

- clindamycin at a dosage of 3x 0.6 g/d (SPC) is not effective enough in severe phlegmons. Therefore, higher doses are recommended for severe skin infections (for example 3x 0.9 g/d, 4x 0.6 g/d or 3x 1.2 g) (expert opinion, the maximum dose according to the package insert is 4.8 g/d).

The higher doses are recommended in the presence of relevant comorbidities, severe progressive infection, incipient sepsis and/or lack of response of the otherwise empirically effective antibiotics. They are usually based on expert opinions, as appropriate dose studies are not available. 

In severe *Staphylococcus aureus* infections without adequate response, a combination of a penicillinase-resistant penicillin with rifampicin, fosfomycin or fusidic acid (no parenteral formulation available in Germany and Switzerland, oral treatment may not always be reliable due to number and size of the tablets) may be considered. Due to rapid development of resistance, however, no monotherapy and no long-term treatment should be carried out using these substances; there are few studies and development of resistance by *Staphylococcus aureus* against these three combination partners is possible under treatment.

- With suspected complicated, chronic, possibly polymicrobial SSTI or suspected involvement of other Gram-positive or Gram-negative pathogens and anaerobes (depends for example on skin region and contamination of possible entry sites):
  - aminopenicillin plus beta-lactamase-inhibitor (amoxicillin/clavulanic acid 3x 2.2 g/d i.v.) OR
  - ampicillin/sulbactam 3x 3 g/d i.v.; OR
  - clindamycin (3x 0.9 g/d up to 3x 1.2 g/d or 3x 1.8 g/d i.v.) in case of strongly suspected Gram-positive pathogens and anaerobes
  - In cases of penicillin allergy, moxifloxacin is recommended [60] (1x 400 mg/d, in the first two days a dose of 2x 400 mg/d may be considered); if necessary combine with fusidic acid (no parenteral formulation available in Germany and Switzerland, the study situation is limited)

The treatment duration usually given is at least 7 days, in some cases up to 21 days for moxifloxacin and sequential therapy [29].

- If severe SSTI with nosocomial origin are suspected with additional infection-related signs of sepsis or criteria of the former SIRS definition in patients with multiple comorbidities (including severe circulatory disorder, chemotherapy for malignancies, neutropenia, severe cell-mediated immunity disorders) and/or additional organ dysfunction:
  - piperacillin/tazobactam in severe SSTIs with Staphylococcus aureus (not MRSA), Gram-negative pathogens and anaerobes (for example, in diabetes mellitus or PAD, including bite injuries) [29], [61]; only in higher doses in immunosuppressed or neutropenic patients.
  - Carbapenems (imipenem; meropenem, ertapenem) in severe phlegmons with Gram-negative pathogens in immunosuppressed or neutropenic patients or in patients with deep, limb-endangering infection (but not by MRSA) in the context of peripheral vasculopathy or severe diabetes mellitus (“diabetic foot”), see below) [62], [63].
    Ertapenem can also be used for treating out-patients because of the once-daily administration; the approved dose is 1 g/d [64], [65]; 2 g/d can be considered. Imipenem/cilastatin is more epileptogenic and less stable than the other carbapenems in prolonged continuous infusions; in severe infections a high initial dose of meropenem (3x 2 g) can be considered (expert opinion).
  - Tigecycline in multidrug-resistant Gram-positive and Gram-negative pathogens such as MRSA (see below), Enterobacteriaceae, *Acinetobacter baumannii*, *Stenotrophomonas maltophilia*, anaerobes; not effective against *Pseudomonas aeruginosa*, *Proteus* spp. and *Morganella morganii*.
- In patients with soft tissue infection, fever and neutropenia (**first** episode) *Pseudomonas aeruginosa*-effective antibiotics:
  - piperacillin/tazobactam plus the addition of piperacillin to achieve a sufficiently high dose of piperacillin (at least 4 4.5 g/d); or piperacillin alone; in severe infections with *Pseudomonas aeruginosa*, continuous infusion may be more effective than administration at intervals [66]; otherwise
  - imipenem/cilastatin or meropenem [12] OR as a second choice ceftazidime (BEWARE: inadequate activity against staphylococci); OR
  - cefepime (BEWARE: comparatively low activity against staphylococci).
- In patients with SSTIs and persistent or recurrent neutropenia with fever
  - antibiotics effective against *Pseudomonas aeruginosa* (see above) PLUS
  - treatment against yeasts and molds (*Candida, Aspergillus, Fusarium*) PLUS
  - vancomycin OR linezolid OR daptomycin OR ceftaroline [12].
- If there is evidence of MRSA or a risk of MRSA infection: (see section on “MRSA”):
  - vancomycin OR linezolid OR another antibiotic effective against MRSA; possibly combined with piperacillin/tazobactam OR a carbapenem if additional pathogens are suspected [12].

If detected or strongly suspected, the following pathogens require specific antibiotics:

- *Aeromonas hydrophila*, for example in traumas involving exposure to fresh water:
  - doxycycline 2x 100 mg/d i.v. PLUS ciprofloxacin 2x 400 mg/d i.v. OR
  - doxycycline 2x 100 mg/d i.v. PLUS ceftriaxone 1x 1–2 g/d i.v.) [12]
- *Vibrio* spp. (*Vibrio vulnificus, Vibrio alginolyticus, Vibrio parahaemolyticus*) in trauma involving exposure to salt water:
  - doxycycline 2x 100 mg/d i.v. PLUS Ceftriaxone 1x 1 g/d i.v. [12]
- *Haemophilus influenzae* in children (periorbital cellulitis):
  - Cefuroxime (children from 2 months up to 14 years: 3x 0.01 g/kg /d to 3x 0.03 g/kg/d; usually a daily dose of 0.06 g/kg body weight/day is enough)

*Comment on the evidence:* Group 4 fluoroquinolones (moxifloxacin) show activity against Gram-positive pathogens (streptococci, staphylococci) and are also active against anaerobes and Gram-negative pathogens but not against *Pseudomonas aeruginosa*. Moxifloxacin was as effective as initial intravenous administration of piperacillin/tazobactam in studies with sequential administration (first i.v., then p.o.), followed by oral administration of amoxicillin/clavulanic acid [29]. However, about 4–10% of patients treated with moxifloxacin have adverse reactions, most commonly in the gastrointestinal tract, nervous system, the vascular system and the skin. In patients with electrolyte imbalances (hypokalaemia) or concomitant use of antiarrhythmic drugs, there is an increased risk of torsade de pointes. Rarely, cases of hepatitis to the point of liver failure have been observed. In any case, there is a strong selection pressure.

- Group 2 fluoroquinolones (for example ciprofloxacin) show very good activity against Enterobacteriaceae, less against *Pseudomonas aeruginosa* and weak to inadequate activity against staphylococci, streptococci, and enterococci [67], [68]. In calculated initial treatment of severe infections, they are important combination partners, if there is a high likelihood of Gram-negative enterobacteria being the cause. In a randomized study of SSTI treatment, ciprofloxacin demonstrated efficacy comparable to that of ceftazidime [69]. Group 3 fluoroquinolones (levofloxacin) show higher activity against Gram-positive pathogens (streptococci, staphylococci) than group 2 fluoroquinolones but poorer activity against *Pseudomonas aeruginosa* [58], [70].
- Amoxicillin/clavulanic acid work well against methicillin-sensitive staphylococci, *Haemophilus influenzae*, *Bacteroides fragilis* and some enterobacteria such as *Escherichia coli*, *Klebsiella pneumoniae* and *Proteus* spp. but not against *Enterobacter* spp., *Serratia marcescens* and *Morganella morganii*. Depending on the dose, it often leads to gastrointestinal problems, the severity of which can be reduced when taken with meals as well as by fractionation of high doses and prior administration of metoclopramide. An increase in transaminases up to 3–4 times the norm can be tolerated if there is no severe liver damage and without occurrence of cholestasis or hepatocellular damage.
- Metronidazole is used against mixed aerobic-anaerobic infections in combination with other antibiotics when isolates that are resistant to clindamycin are involved (in particular *Bacteroides fragilis*).
- The oxazolidinones linezolid and tedizolid only show activity against Gram-positive bacteria (including MRSA and VRE). Their use should be limited to (targeted) treatment of infections caused by multidrug-resistant Gram-positive pathogens. Linezolid shows very good distribution in skin or soft tissue and works well orally and parenterally observing adverse effects and carrying out check-ups. Tedizolid (once daily orally or parenterally) caused fewer gastrointestinal complaints and thrombocytopenia than linezolid at 6 days of treatment [71], [72], [73].
- Piperacillin/tazobactam has a relatively broad action spectrum and is reserved for the treatment of severe SSTIs suspected of involving *Staphylococcus aureus*, Gram-negative pathogens and anaerobes (i.e. infections in patients with diabetes mellitus, PAOD or bite injuries). In infections with *Pseudomonas* spp. the dose should be at least 4x 4.5 g/d. For the most severe SSTIs (Fournier gangrene) up to 4x 9 g can be used. Publications only cover doses up to 4x 4.5 g but high doses are possible due to its wide therapeutic range. In severe infections with *Pseudomonas aeruginosa*, continuous or prolonged infusion may be more effective than administration at intervals [66].
- Cephalosporins of group 3: Ceftazidime (group 3b), in contrast to cefotaxime and ceftriaxone (group 3a), has good activity against *Pseudomonas* but does not show sufficient activity against staphylococci. The effect of cefotaxime and ceftriaxone on staphylococci is weaker than that of group 1 and 2 cephalosporins. In a randomized study on the treatment of SSTI patents, ceftazidime was as effective as ciprofloxacin [69], [74].
- Group 5 parenteral cephalosporins (ceftobiprole and ceftaroline) have a broad action spectrum that includes Gram-positive pathogens (including MRSA) as well as many Gram-negative pathogens but not ESBL-producing Enterobacteriaceae and not *Acinetobacter baumannii*. Ceftobiprole, in contrast to ceftaroline, also shows activity against *Pseudomonas aeruginosa*. Their use is indicated for MRSA infections. The efficacy of monotherapy with ceftobiprole was comparable to that of vancomycin plus ceftazidime in patients with complicated SSTIs caused by Gram-positive and Gram-negative bacteria [75].
- Tigecycline has a broad spectrum of activity and also covers many multidrug-resistant Gram-positive and Gram-negative pathogens such as MRSA, ESBL-producing Enterobacteriaceae, *Acinetobacter baumannii*, *Stenotrophomonas maltophilia*, anaerobes but not *Pseudomonas aeruginosa*, *Proteus* spp. and *Morganella morganii*. In complex skin and soft tissue infections, it has shown efficacy comparable to the fluoroquinolone delafloxacin [76] or ampicillin/sulbactam or amoxicillin/clavulanic acid in two studies [28]. However, in a study on the treatment of diabetic patients with infected feet it was inferior to ertapenem [77].
- Carbapenems (all parenteral) have a very broad antimicrobial spectrum. They cover most Gram-positive pathogens, including penicillinase-producing staphylococci (but no MRSA), many Gram-negative bacteria, including anaerobes and extended-spectrum beta-lactamase-producing pathogens. But they are ineffective against *Stenotrophomonas maltophilia*, *Clostridium difficile* and enterococci. Ertapenem also has inadequate activity against *Pseudomonas aeruginosa* and *Acinetobacter baumannii*. Carbapenems are indicated for the treatment of severe phlegmons with Gram-negative pathogens in immunosuppressed patients or in patients with deep limb-endangering infections in the context of peripheral vasculopathy or severe diabetes mellitus (“diabetic foot”, see below) [63]. Ertapenem has the advantage of once-daily administration due to the long half-life, which makes it attractive for long-lasting out-patient parenteral antibiotic treatment. In MSSA, it has comparable efficacy to piperacillin/tazobactam. The approved dosage is 1 g/d [64], [65], [77], [78] but 2 g/d can be considered. If patients are at an increased risk of MRSA, carbapenems should be given in combination with vancomycin or linezolid (see below, section on MRSA).

The glycopeptides (vancomycin, teicoplanin), lipoglycopeptides (dalbavancin, oritavancin), lipopetides (daptomycin), oxazolidinones (linezolid, tedizolide) [71] (for more references see the section on MRSA below) and group 5 MRSA-active cephalosporins (ceftobiprole, ceftaroline) should only be used in cases of suspected MRSA infection, once an antibiogram is available or if other substances are not suitable. Therefore they are not all listed in Table 1 (Tab. 1) (for example teicoplanin or ceftobiprole).

In comparative studies and in meta-analyzes, vancomycin has proven to have inferior clinical and microbiological efficacy [79], [80] than linezolid and telavancin (in MRSA) but telavancin has more serious adverse effects and carries the risk of nephrotoxicity, daptomycin that of CK increase, and linezolid that of thrombocytopenia. On the basis of the (comparative) studies to date, there was no overall reason to prefer the newer substances to vancomycin in general [79], [80], [81], [82], [83].

There are only a few studies on the specific indications listed in Table 1 (Tab. 1), such as severe soft tissue infections in patients with neutropenia and fever or additional persistent or recurrent fever neutropenia. The IDSA [12] et al. however have developed guidelines regarding these and to which we refer. A French comparative study with cefepime and ceftazidime, each in combination with amikacin, has shown good results in neutropenic patients with febrile episodes [84].

Duration of treatment should be based on the clinical response. In general, it is 5–10 days for uncomplicated infections and 7–14 days for immunocompromised patients [13].

### Foot infections in diabetes mellitus

Foot lesions in diabetics arise as a result of complex neuropathic and angiopathic late damage. The diminished immune response often leads to painless soft tissue infections after trivial trauma and permanent mechanical stress, which can spread to adjacent tendons, joint capsules and bones or to the entire foot. According to the previous criteria of the FDA it is almost always a complicated soft tissue infection [5]. 

Foot infections of patients with severe diabetes mellitus should first be classified according to severity, for instance according to an international classification such as PEDIS (an acronym for **P**erfusion, **E**xtent/size, **D**epth/tissue loss, **I**nfection, and **S**ensation). The choice of antibiotic should then be in accordance with the recommendations of the International Working Group on Diabetic Foot. A differentiation between very superficial and deeper but clinically moderate infections is particularly useful because of the different pathogen spectrum [85], [86], [87], [88]. Depending on the severity, additional relevant comorbidities and suspected polymicrobial infection, the recommendations of the International Working Group on Diabetic Foot are similar to our above-mentioned recommendations for the treatment of limited or severe phlegmons. PEDIS 1 (non-infected ulcers) do not require antibiotics.

- For PEDIS 2 (= ulcer, superficial infection, ≤2 cm diameter):
  - cefuroxime OR cefazolin (or if appropriate oral treatment with, for example, cefalexin),
  - if a broad pathogen spectrum cannot be ruled out: aminopenicillin plus a beta-lactamase inhibitor [61], [85], [86], [87], [88], [89], [90],
  - in case of penicillin allergy: moxifloxacin.

*Comment:* The most common pathogens are *Staphylococcus aureus* and beta-hemolytic streptococci but since a mixed infection may be present, the action spectrum of the antibiotic used should also cover other likely pathogens. There are studies on the use of aminopenicillins plus beta-lactamase inhibitors which allow a higher evidence level [61], [85], [86], [87], [88], [89], [90] but their action spectrum for this indication is very broad (expert opinion), so that at least in cases of PEDIS 2 initially one of the above cephalosporins can be swapped for an aminopenicillin plus beta-lactamase inhibitor in case of insufficient response. Evidence is also better for moxifloxacin but because of the slightly less favorable risk-benefit ratio (see comment in the section on severe phlegmons), we see it as a good substitute in patients allergic to penicillin.

- In PEDIS 3 (= ulcer with deep infection): Aminopenicillin plus beta-lactamase inhibitor OR moxifloxacin OR piperacillin/tazobactam [29], [61] OR ciprofloxacin or levofloxacin plus metronidazole (to cover anaerobes) OR carbapenems

*Comment:* The most common pathogens besides *Staphylococcus aureus* and beta-hemolytic streptococci are Enterobacteriaceae and anaerobes. The pathogens should be tested for sensitivity and the antibiotic dose adjusted (and de-escalated if appropriate) which allows the selection of antibiotics which will achieve sufficiently high levels of efficacy in soft tissue and adjacent bone regions (for example clindamycin, fluoroquinolones, fosfomycin). 

### Severe (toxin-related) life-threatening necrotizing soft tissue infections 

The so-called necrotizing soft tissue infections with an immediate threat to life (for example necrotizing fasciitis) constitute a distinct group with particular toxin-mediated pathogenesis. They are grouped together under the name “necrotizing skin and soft tissue infections” (nSSTI) in the UK and the US [3]. They are not on a direct continuum with the aforementioned soft tissue infections (phlegmons, abscesses). 

They affect the skin, subcutaneous tissue, the fascia including the underlying muscle (necrotizing fasciitis) and skeletal muscle (clostridial, necrotizing myositis = gas gangrene) or non-clostridial, mostly streptogenic myonecrosis or pyomyositis. They are characterized in their pathophysiology by the effects of bacterial toxins, intravascular thrombosis, ischemic necroses and by a malfunction of humoral and cellular host defense. Pathogens entry sites are, in addition to hematogenous scattering, mostly trvial traumas, infected surgical wounds and injection sites (injection abscess), less frequently inflamed periurethral glands or perianal infections (Fournier gangrene). 

In about 80% of cases (type I), these are mixed infections caused by Gram-positive pathogens (streptococci, sometimes also staphylococci, etc.), anaerobes (*Bacteroides fragilis, Prevotella melaninogenica*) and Enterobacteriaceae [91], [92]. Monoinfections occur in about 20% of cases (type II); they are predominantly found on the extremities and are usually triggered by toxin-producing group A hemolytic streptococci with certain M-proteins, in recent years increasingly from the hyperinvasive serotype M1T1 or, more rarely, by mostly PVL-producing *Staphylococcus aureus*. Less common are type III (after consumption of seafood or through water-contaminated wounds caused by *Vibrio* spp. and *Aeromonas* spp.) and type IV (after trauma, caused by zygomycetes and other fungi).

Fournier gangrene is the rapidly progressing polymicrobial necrotizing fasciitis of the perineum, scrotum and penis in men but can also occur perineally and genitally in women and children. It may extend further into the anorectal region, to the thighs and to the abdominal wall, and includes occlusion of the subcutaneous arteries, necrosis of the skin, subcutis, muscles, fasciae with pus and often gas formation. There are differences in the pathogen spectrum between the urogenital and rectal forms of Fournier gangrene. *Staphylococcus aureus* is more often involved in the urogenital form (for example, after severe urinary tract infections) but not usually in anal manifestations.

Characteristic of the severe, toxin-related necrotizing soft tissue infections is the acute foudroyante progression with severe general and ultimately shock symptoms or an early onset of organ failure. The extreme, ischemia-related, local pain, which is in no apparent relation to the initially visible clinical findings, is both the leading and only typical early symptom 

If suspected, early surgical inspection is recommended but the following lab tests may help distinguish it from non-necrotizing phlegmons: Leukocytosis >15,400 cells/mm^3^ or sodium i.S: >135 mEq/L (sensitivity 90%, specificity 76%) [93] or lactate of 2 mmol/L (sensitivity 100%, specificity 76%) [13], [94]. Imaging procedures do not have a high sensitivity for early diagnosis but gas accumulation in CT without trauma or with a fluid level (→ abscess) may be a characteristic, although not obligatory, sign [13].

Starting treatment early and a sufficiently high dose of antibiotics from the start in the maximum doses [16], [17] are key for the prognosis. Immediate measures must therefore be taken without delay. They include radical surgical debridement with complementary antibiotic treatment and intensive care started at the latest during surgery. With regard to calculated initial treatment, it is important to cover likely Gram-positive and Gram-negative bacteria (including anaerobes) as well as the inhibition of toxin formation and beta-lactamase activity with substances that have good tissue permeability. The relevant substances should be given in sufficiently high doses.

## Antibiotics of first choice for initial calculated treatment

Acylaminopenicillin plus beta-lactamase inhibitor (for example piperacillin/tazobactam) or a carbapenem of group 1 or group 2 (each in high doses) in combination with clindamycin or linezolid are both effective against both polymicrobial and streptococcal or CA-MSSA-related necrotizing infections. 

If MRSA is suspected, linezolid should be used as a combination partner. The additional administration of linezolid or clindamycin is not only recommended for this reason but also to reduce toxic effects of Gram-positive bacteria by inhibiting protein biosynthesis. In this way septic complications can be alleviated by exotoxin production (for example superantigens) [42], [95]. It should be emphasized that there is no evidence from controlled clinical trials as yet on the additional use of a protein biosynthesis inhibitor but only data from animal tests (*Clostridium-perfringens* infection in mice [12], [95]).

Alternatively, use of a group 3 cephalosporin with metronidazole is an option. We don’t favor the primary therapy with vancomycin recommended in the IDSA guidelines because, on the one hand the pathogen situation in the USA is different (about 60% of all soft tissue infections are caused by CA-MRSA, compared to only 1–3% in Germany) and because patients often either suffer from pre-operative compensated renal insufficiency or significant renal impairment as a result of advanced sepsis which would be worsened by the administration of (high-dose) vancomycin. In case of treatment failure of the above-mentioned first-choice substances tigecycline can be used in monotherapy or combination therapy in the sense of a so-called second-line option [96]. Since in the genito-urinary form of Fournier gangrene *Staphylococcus aureus* may also be involved as part of a mixed infection, a cephalosporin effective against staphylococci can be used instead of piperacillin/tazobactam [96], especially following pathogen detection.

As duration of treatment we recommend 7–10 days after adequate surgical restoration (the guideline of the Infectious Diseases Society of America recommends 2–3 weeks [12]).

**If certain pathogens are suspected: **

- Clostridial gangrene or myonecrosis
  1. Surgical debridement of all affected tissue
  2. prior to pathogen identification: vancomycin plus piperacillin/tazobactam OR ampicillin/sulbactam OR carbapenem. **Following** pathogen identification of Clostridia: penicillin plus clindamycin [12]
- Streptococci of group A
  1. Surgical debridement of all affected tissue and
  2. penicillin plus clindamycin
- Aeromonas hydrophila, Vibrio vulnificus
  1. Surgical debridement and
  2. for example doxycycline plus ceftriaxone

The use of hyperbaric oxygen (HBO) is controversial in the literature. In some groups, it was possible to reduce the necessary debridement in some patients; in other publications this effect did not appear. Since it was not possible to significantly reduce mortality through HBO, the general use of HBO is currently not recommended in reviews [12].

### Therapy of skin and soft tissue infections with MRSA as a suspected pathogen

If MRSA is suspected, penicillins, group 1–4 cephalosporins, beta-lactam/beta-lactamase inhibitor combinations and carbapenems are not an option for calculated antibiotic treatment. 

Regarding MRSA-effective cephalosporins of group 5 (ceftobiprole, ceftaroline), there are a limited number of clinical studies.

If HA-MRSA is suspected as a pathogen, its multi-resistance should be considered. By contrast, CA-MRSA and LA-MRSA have significantly less resistance to other classes of antibiotics (exceptions: tetracycline resistance in LA-MRSA, fusidic acid resistance in CA-MRSA). Additional information on MRSA can be found in chapter 2 [97].

HA-MRSA are almost always resistant to fluoroquinolones (approx. 85–90%) and approx. 50–70% to clindamycin and erythromycin. Resistance to doxycycline is found in 5–7% of HA-MRSA strains, and <2% of strains each have resistance to trimethoprim/sulfamethoxazole, fosfomycin and rifampicin. Isolates with resistance to daptomycin, linezolid and tigecycline are very rare (current resistance data, see https://www.p-e-g.org/resistenzdaten.html and https://ars.rki.de/).

In Germany and Austria generally HA strains can be assumed as pathogens in suspected MRSA infections. Overall, LA and CA-MRSA strains are still rare. However in regions with intensive agricultural animal husbandry (northwest Germany), the proportion of LA-MRSA strains in all MRSA can be significantly higher [37].

- *First choice:* Linezolid [81], [82], [83] OR daptomycin [98], [99].
- *Second choice, for instance when preferred antibiotic does not work:* Vancomycin with a combination partner (rifampicin or fosfomycin or fusidic acid).

The following is recommended for uncomplicated SSTIs if CA-MRSA is suspected in countries with high CA-MRSA prevalence: Trimethoprim/sulfamethoxazole (oral, 2x 160/800 mg/day), which is said to be active against CA-MRSA but also against HA-MRSA and streptococci) [30], [31], [32], [33] OR clindamycin which is also active against streptococci (oral or parenteral, in studies 3x 300 mg/day [34] but we recommend higher doses) OR doxycycline (2x 0.1 g p.o.) [12], [100].

*Comment on the evidence:* A rigorous analysis by the Cochrane Initiative of the antibiotic treatment of infected wounds and soft tissue infections by MRSA revealed no clear advantage of linezolid over vancomycin due to an insufficient number of randomized comparative studies [101]. However, in two studies, treatment and hospital stay were shorter under treatment with linezolid than vancomycin [82], [83] and a recent review that looked at 21 meta-analyzes concludes that from a clinical and microbiological viewpoint linezolid, such as teicoplanin, offers advantages over vancomycin in dermal and soft tissue infections caused by MRSA but should not, due to its potential adverse effects, be preferred in general [81]. The changes in the blood count observed under treatment with linezolid or the peripheral neuropathy and/or optic neuropathy predominantly occur after treatment duration of more than 28 days. In a complicated SSTI, daptomycin can also be used [98], [99]. In meta-analyzes, it showed efficacy which was not inferior to vancomycin in MRSA infections and a relatively good safety profile [89], [99]. 

Our recommendation grade for linezolid and daptomycin is similar but for linezolid the evidence is greater. Daptomycin is now generally recommended in SSTIs, with the approved dose of 6 mg/kg/d in the presence of MRSA bacteremia and 8–10 mg/kg/d in infections which are difficult to treat, instead of the 4 mg/kg/d, which is often used in studies [99], [102]. Daptomycin showed good efficacy in complicated soft tissue infections in an observational study (EU-CORE) [103]. There are no comparative clinical studies on linezolid. 

Tedizolid was not inferior to linezolid in the two pivotal comparative studies. However the duration of treatment with linezolid was 6 instead of 10 days, [71], [73].

Dalbavancin (1 g on day 1 and 0.5 g on day 8) showed no inferiority to vancomycin (2x 1 g/day for at least 3 days, optional sequential therapy with 2x 0.6 g linezolid p.o. for a total of 10–14 days) in a comparative study for effective suppression of infection spread and fever after 48–72 hours but as it only required two administrations, dalbavancin was easier to handle [104], [105].

Tigecycline has been shown to be equivalent to ampicillin/sulbactam in severe infections but is inferior to ertapenem in the treatment of foot infections (with and without osteomyelitis) in patients with severe diabetes mellitus [28], [77]. However tigecycline is an alternative in the treatment of polymicrobial infections with MRSA involvement (for instance infected diabetic foot syndrome type PEDIS 3 or 4). 

If a glycopeptide (vancomycin or teicoplanin) is given, combination with rifampicin or fosfomycin is recommended. Fosfomycin reaches sufficiently high concentrations in soft tissue and bone [106].

CA-MRSA are usually sensitive to clindamycin, cotrimoxazole, doxycycline and often also to fluoroquinolones. CA-MRSA infections, particularly in the US, are found in groups of healthy patients at increased risk of skin injury and sharing toiletries (military, prison, sports clubs). Due to their toxin production (panton-valentine-leukocidin, PVL), the clinical spectrum ranges from boils to necrotizing fasciitis [107].

A study conducted in the US found that trimethoprim/sulfamethoxazole (2x 320 mg/1,600 mg/day for 7 days) in drained abscesses increased the healing rate compared to a placebo (92.9% versus 85.7%) and reduced the number of repeat drains or further abscesses [31]. In this study, PVL-positive MRSA (USA300) was detected in over 40% of abscesses. The already mentioned comparative studies on SSTIs (including abscesses) showed no significant differences between trimethoprim/sulfamethoxazole and clindamycin [34] or between different doses of trimethoprim/sulfamethoxazole for 7–5 days each [35].

In the PEG resistance study in 2013, all HA-MRSA (n=75) and *Streptococcus pyogenes isolates* (n=246) were also cotrimoxazole-sensitive [32], [33].

The implementation of the KRINKO recommendations for the prevention and control of MRSA is essential to prevent the spread of MRSA within medical and nursing facilities [36]. For dermatological lesions, wound closure should be the goal in colonization or infection with MRSA.

### Bite wounds

Bite injuries can lead to severe mechanical tissue destruction, which can subsequently cause severe infection by contamination with the oral microbiota of the biting subject. Even bite injuries which appear insignificant at first glance should not be underestimated, since the extent of the injury can be obscured by the sometimes relatively small lesions on the skin surface. Especially in case of animal bites, the microbiological lab should be informed, since microorganisms which do not usually appear on the human-adapted spectrum must also be taken into account. Bite wounds require a multidisciplinary approach [108].

In cats and dogs these are mostly aerobic-anaerobic mixed infections involving *Pasteurella multocida*, *Capnocytophaga* spp., *Bartonella* spp., *Staphylococcus aureus* and other coagulase-positive staphylococci (for example members of the Staphylococcus pseudintermedius group), beta-hemolytic streptococci and anaerobes. The transmitted pathogens reach deeper tissue layers relatively easily due to the punctiform nature of the bites, especially in cat bites. If bones or tendons are affected, this can lead to chronic osteomyelitis or tendomyositis or tendosynovitis. Among the most commonly isolated anaerobes for cat and dog bites are *Bacteroides* spp., *Fusobacterium* spp., *Porphyromonas* spp., *Prevotella* spp., *Propionibacterium* spp. and *Peptostreptococcus* spp.

In rat bites, *Streptobacillus moniliformis*, the causative agent of rat bite fever, must be considered.

Bite injuries by humans lead to both acute and chronic-recurrent infections. Most of them are Gram-positive (usually *Streptococcus* spp. and *Staphylococcus* aureus) and Gram-negative pathogens (for instance *Pasteurella multocida* and other *Pasteurella* species, *Mannheimia* [formerly *Pasteurella]* *haemolytica*, *Haemophilus* spp., *Eikenella corrodens*) and anaerobes, including *Fusobacteria*, *Prevotella* and *Porphyromonas* species.

Increasingly, MRSA and methicillin-resistant (MR), coagulase-positive non-Staphylococcus aureus staphylococcal isolates (MR-Staphylococcus pseudintermedius) are found in companion (dog, cat, horse) and food-producing animals (pig, cattle, poultry).

For all bite injuries, the tetanus vaccination status must be checked and the risk of infection with rabies determined.

*Immediate measures:* There are no evidence-based guidelines for treatment to date. According to a review by Rothe et al. including the readers’ discussion of this article, [108], optimal surgical management and specialized care are key to the management of wound infections. Correct necrosectomy, a mechanical reduction of the number of germs and the optimization of the microcirculation in the wound area form the basis of surgical treatment. Since in complicated cases severe infections almost always originate from the subcutaneous fascial structures (amongst others, tendon tissue and joint capsules), surgical exploration should include the fascia [109]. The use of wound irrigation for the mechanical cleansing and pathogen reduction of bites is controversial. They are no replacement for necrosectomy. The fact that both wound cleansing and irrigation belong to the generally accepted measures of wound treatment and can lead to a reduction of the pathogen load speaks for irrigation. An argument against irrigation is that it may carry pathogens into other tissue areas and that there is no evidence for this measure. A working group of the German Society for Hospital Hygiene recommends a combined approach with surgical debridement and antiseptic lavage depending on the age and type of wound.

Primary wound closure is not recommended, with one possible exception being facial bites; especially in this case, however, the wound must be irrigated and explored sufficiently and debrided or necrosectomized and a prophylactic administration of antibiotics given. However, even in facial wounds we recommend irrigating the wound for 2–3 days, leaving it open and only then closing it to reduce the risk of infection; secondary wound closure still leads to good cosmetic results [110], [111], [112].

The following proposal was submitted for severity level classification of bite injuries [108]:

- Grade I: superficial skin lesion, lacerated wound, scratch wound, bite duct, contusion
- Grade II: skin wound, reaching fascia/musculature/cartilage
- Grade III: wound with tissue necrosis or substance defect

For open dog bite injuries in the facial area, the following stages were proposed [113]:

- Stage I: superficial injury without involvement of the musculature
- Stage II: deep injury with involvement of the musculature
- Stage III: deep injury with involvement of the musculature and substance defect
- Stage IVA: Stage III with vascular and nerve injury
- Stage IVB: Stage III with bone involvement

The indications for (prophylactic) antibiotic administration are:

- moderate to severe and deep bites
- bite marks on the hand and face
- bite marks, possibly reaching to periosteum or joint capsule
- immunosuppression/immunodeficiency
- hepatic insufficiency
- condition after splenectomy
- edema in the affected area

Every human and animal bite is highly likely to represent a potential risk of infection due to the very high number of microorganisms transmitted through the teeth and saliva, with the added problem of depth and extent of and tissue trauma not always being recognized in routine medical practice. Therefore, as a rule, we also recommend prophylactic treatment with antibiotics, if depth and extent of the bite wound cannot be assessed with certainty. This emphatically applies to the indications to which other authors or recommendations limit themselves (see below) [110], [111], [112].

Prophylactic antibiotics for cat and dog bites: Aminopenicillin plus beta-lactamase-inhibitor. Prophylactic administration should last for 3–5 days.

### Infected bite marks 

Purulent bites are usually polymicrobially colonized, not obviously purulent infections are more likely to be colonized by staphylococci and streptococci. *Pasteurella* species are found in both purulent and non-purulent infections [114]. 

Depending on the animal and extent of the bite, the following calculated administration of antibiotics is recommended:

*First choice* with cats and dogs: Aminopenicillin plus beta-lactamase-inhibitor.

*Second choice* (depending on the animal and extent): Cefuroxime PLUS clindamycin OR metronidazole against anaerobes for deep bites, OR moxifloxacin [109], [115] PLUS clindamycin or metronidazole [12] OR piperacillin/tazobactam (and treatment based on an antibiogram as soon as possible) [12].

If MRSA/MR *Staphylococcus pseudintermedius* is suspected, MRSA-effective antibiotics should be included.

For human bites: Aminopenicillin plus beta-lactamase inhibitor OR ertapenem, amongst other things because of *Eikenella corrodens* [12]; in severe infections or immunosuppressed patients piperacillin/tazobactam. 

*Comment on the evidence:* Currently available studies on prophylactic antibiotic treatment in bite wounds are not consistent and do not allow a clear statement. The benefit seems to be marginal immunocompetent patients with relatively shallow bites inflicted by a dog 12–24 hrs ago [110], [111]. A Cochrane analysis recommends prophylactic treatment only for bites on the hand and for human bites. However, only a few clinical studies were available [112]. As the depth and extent of the bite wound cannot always be properly assessed by appropriate probing (which should be performed down to the fascia), however, we recommend expanding the indication for a short antibiotic prophylaxis to all cases where the above-mentioned criteria cannot all be excluded beyond doubt.

The recommendations for bites which are already infected are based primarily on the microbiological findings [114] and individual studies [12], [114], [115].

In the American Practice Guidelines for the Diagnosis and Management of Skin and Soft Tissue Infections, aminopenicillin plus beta-lactamase inhibitor and ertapenem are recommended for the treatment of infected human bites. The antibiotics mentioned have activity against *Eikenella corrodens*, anaerobes and streptococci but have no activity against MRSA [12]. In contrast, piperacillin/tazobactam is not recommended in the Practice Guidelines but due to its action spectrum it is well-suited for the treatment of severe infections caused by *Staphylococcus aureus* (MSSA only), Gram-negative pathogens and anaerobes, especially in immunosuppressed patients (high dosage possible) following bite injuries.

## Notes

This is the ninth chapter of the guideline “Calculated initial parenteral treatment of bacterial infections in adults – update 2018” in the 2^nd^ updated version. The German guideline by the Paul-Ehrlich-Gesellschaft für Chemotherapie e.V. (PEG) has been translated to address an international audience. 

Following the publication of the 1^st^ version of the guideline in German, these dosage suggestions were updated by the working group: Additional comments on the dosages of penicillin G and penicillin V in the treatment of complicated erysipelas (in the text and in Table 2 (Tab. 2)). 

## Competing interests

The authors declare that they have no competing interests.

## Figures and Tables

**Table 1:**
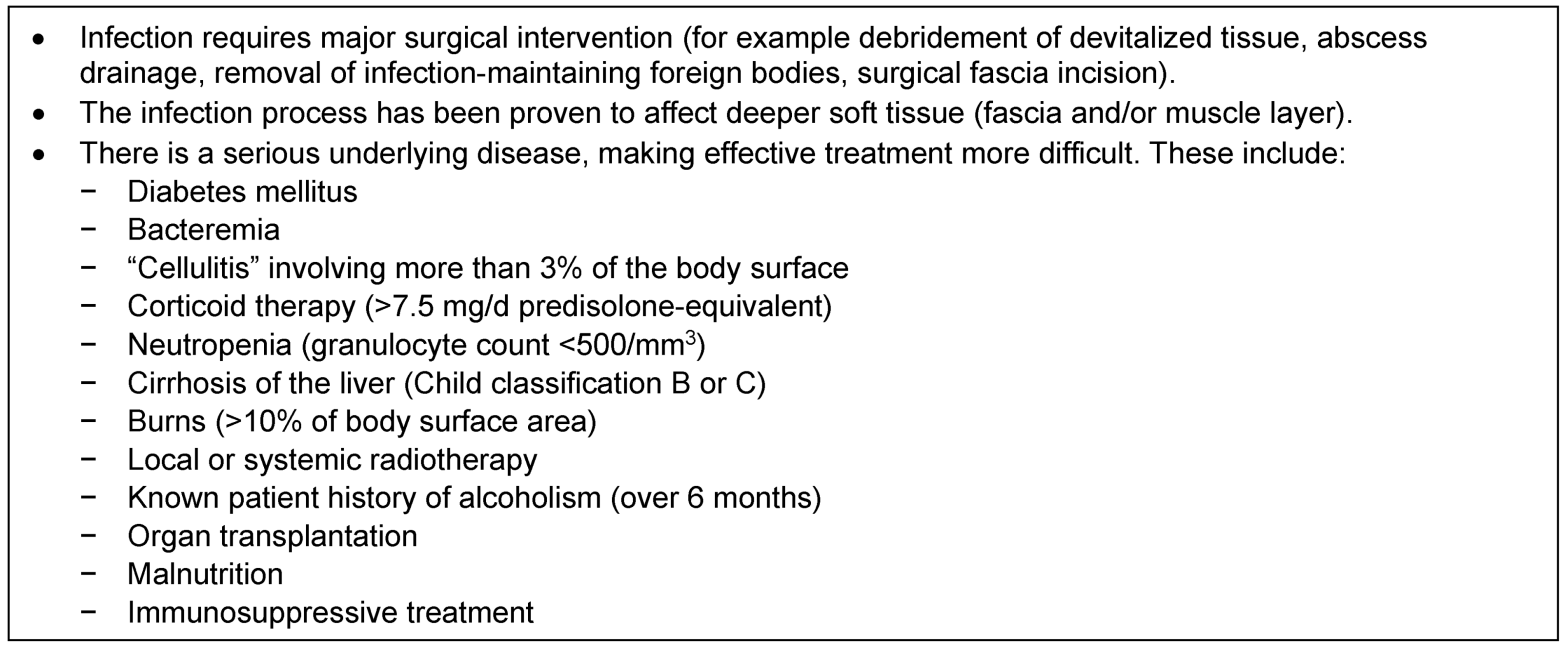
Definition of “complicated skin/soft tissue infection” according to FDA [5], [12], [42]

**Table 2:**
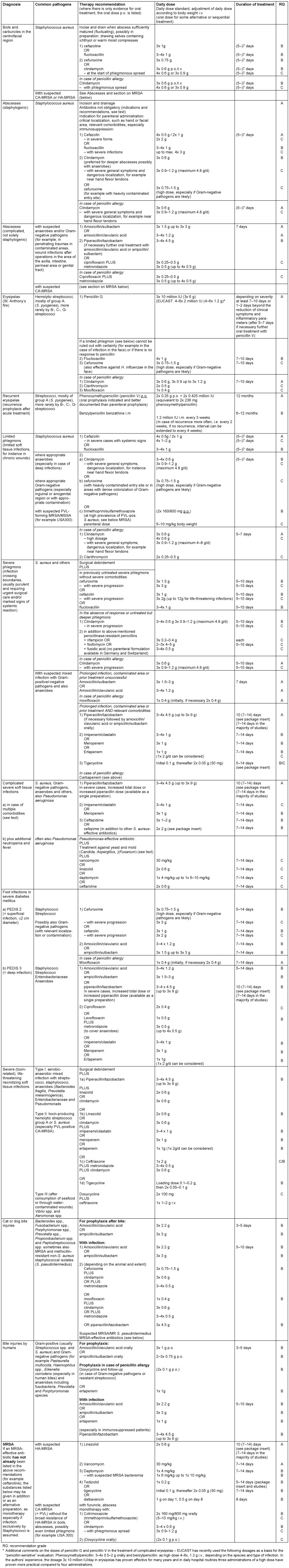
Treatment recommendation tables
